# The Efficacy of Anti-PD-L1 Treatment in Melanoma Is Associated with the Expression of the ECM Molecule EMILIN2

**DOI:** 10.3390/ijms22147511

**Published:** 2021-07-13

**Authors:** Albina Fejza, Maurizio Polano, Lucrezia Camicia, Evelina Poletto, Greta Carobolante, Giuseppe Toffoli, Maurizio Mongiat, Eva Andreuzzi

**Affiliations:** 1 Division of Molecular Oncology, Department of Research and Diagnosis, Centro di Riferimento Oncologico di Aviano (CRO) IRCCS, 33081 Aviano, Italy; albina.fejza@cro.it (A.F.); lucricamicia@gmail.com (L.C.); evelina.poletto@cro.it (E.P.); greta.carobolante@cro.it (G.C.); 2 Experimental and Clinical Pharmacology Unit, Centro di Riferimento Oncologico di Aviano (CRO) IRCCS, 33081 Aviano, Italy; mpolano@cro.it (M.P.); gtoffoli@cro.it (G.T.)

**Keywords:** melanoma, extracellular matrix, tumor microenvironment

## Abstract

The use of immune checkpoint inhibitors has revolutionized the treatment of melanoma patients, leading to remarkable improvements in the cure. However, to ensure a safe and effective treatment, there is the need to develop markers to identify the patients that would most likely respond to the therapies. The microenvironment is gaining attention in this context, since it can regulate both the immunotherapy efficacyand angiogenesis, which is known to be affected by treatment. Here, we investigated the putative role of the ECM molecule EMILIN-2, a tumor suppressive and pro-angiogenic molecule. We verified that the EMILIN2 expression is variable among melanoma patients and is associated with the response to PD-L1 inhibitors. Consistently, in preclinical settings, the absence of *EMILIN-2* is associated with higher PD-L1 expression and increased immunotherapy efficacy. We verified that EMILIN-2 modulates PD-L1 expression in melanoma cells through indirect immune-dependent mechanisms. Notably, upon PD-L1 blockage, *Emilin2^−/−^* mice displayed improved intra-tumoral vessel normalization and decreased tumor hypoxia. Finally, we provide evidence indicating that the inclusion of EMILIN2 in a number of gene expression signatures improves their predictive potential, a further indication that the analysis of this molecule may be key for the development of new markers to predict immunotherapy efficacy.

## 1. Introduction

Tumor escape from the immune system is a tactical approach exploited by transformed cells to avoid being killed, and, to this end, they engage different strategies to achieve the goal. For instance, tumor cells can induce immune suppression, triggering the expression of immunosuppressive molecules, such as the programmed death-ligand 1 (PD-L1) or its receptor programmed death-1 (PD-1), which can inhibit the activation of effector T lymphocytes, ultimately leading to tumor immune escape [1]. Indeed, the employment of immune checkpoint inhibitors in the treatment of melanoma, as well as for other types of patients, has been a clinical breakthrough with a significant improvement inoutcomes [2,3,4]. However, althoughtreatment with anti-PD-1/PD-L1 inhibitors yields unprecedented clinical effects, a consistent number of patients ranging from 40% to 60% are refractory to the therapy and/or experience severe adverse events [5]. Multiple studies have investigated the levels of PD-L1 expression and the activity of intratumoral immune effector cells as predictive biomarkers of therapy response, yet they have failed to efficiently identify the fraction of patients which do not respond to the treatments [6,7]. Thus, there is the need to better clarify the molecular mechanisms regulating the PD-1/PD-L1 pathway and to develop new prognostic and predictive biomarkers.

PD-L1 is widely expressed in the tumor microenvironment not only by lymphocytes, but also by a plethora of other cell types including endothelial cells, cancer-associated fibroblasts (CAFs), mesenchymal stem cells, macrophages and keratinocytes. Moreover, tumor cells themselves often display abnormally high expression of PD-L1, a feature which is considered instrumental to escape from the immune response [1]. The role of the microenvironment in tumor growth and metastasis has long been recognized and, recently, it has been highlighted as a key component in the modulation of the PD-L1 levels [1]. The importance of the tumor microenvironment (TME) in this context is further highlighted by the fact that the angiogenic as well as the hypoxic status of the tumor are key in determining the efficacy of checkpoint inhibition [5]. Indeed, emerging evidence indicate that angiogenesis and immunosuppression occur simultaneously in response to diverse stimuli during tumor development. Within the TME, the interplay between tumor vessels and pro-tumoral immune cells engages a vicious cycle that promotes tumor progression, where the hypoxic conditions within the lesion foster the evasion of tumor cells from immune surveillance, as well as promoting angiogenesis [8]. Importantly, tumor-associated vessels are strikingly abnormal and characterized by diffuse tortuosity, poor pericyte coverage and high permeability, providing favorable routes for metastatic dissemination [9]. For these reasons, the targeting of the tumor vasculature in combination with immune therapies represents an emerging strategy to improve the treatment of melanoma, as well as other malignancies [10,11].

The extracellular matrix (ECM) is recognized as a crucial microenvironmental component affecting both the immune and the angiogenic response [12]. Indeed, recent evidence has indicated that some ECM components, such as collagens and hyaluronan, directly regulate the expression of checkpoint molecules [13] and are associated with the efficacy of PD-1/PD-L1 blockage [14]. Another ECM molecule modulating the TME in melanoma is EMILIN-2 [15,16]. EMILIN-2 exerts a suppressive function in a number of tumor types [17,18] and its expression is often down-regulated during tumor progression [19]. In melanoma, EMILIN-2 promotes angiogenesis by triggering IL-8 expression via the EGF/EGFR pathway, affecting vessel development and functionality [20]. Indeed, in both preclinical settings as well as in patients, EMILIN-2 loss results in reduced chemotherapy efficacy. Recently, EMILIN-2 emerged as the sole ECM molecule whose expression was altered in chemo-resistant colorectal cancer cells, further suggesting the crucial role of this protein in regulating the therapeutic responses [21].

Given these observations, in this study, we aimed to verify if the expression of EMILIN2, which is known to be altered in tumors [22,23], impacts the efficacy of the treatment of melanoma patients with anti-PD-1/PD-L1 agents. We discovered that the expression of EMILIN2 inversely correlates with the efficacy of anti-PD-L1 treatment, additionally underlying the importance of the tumor microenvironment in this context. In addition, we found that the therapeutic blockage of PD-L1 exerts an important effect on vascular normalization, further highlighting the interconnection between immunity and angiogenesis. Taken together, these results suggest the possibility of developing new prognostic and/or predictive markers of immunotherapy efficacy.

## 2. Results

### 2.1. EMILIN2 Expression Is Epigenetically Modulated in Melanoma and Is Associated with the Patient Outcome

**Figure 1:**
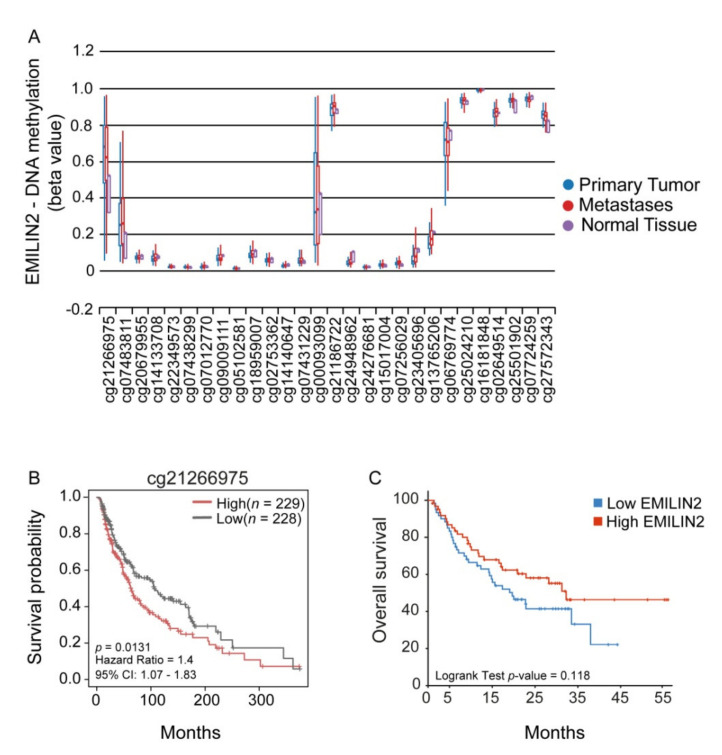
The methylation of the EMILIN2 gene correlates with the overall survival of melanoma patients. (**A**) Evaluation of the methylation levels relative to 28 CpGs of the EMILIN2 gene in normal skin, melanoma and metastases assessed in the TCGA Melanoma cohort (*n* = 477). (**B**) Kaplan–Meier curve reporting the overall survival of the melanoma patients in relation to the methylation of the EMILIN2 gene at cg21266975 site. (**C**) Kaplan–Meier curve stratified according to high and low EMILIN2 mRNA expression levels in a cohort of melanoma patients (Liu et al., 2019). Kaplan–Meier curves were computed using R (version 3.6.1) with *survival* and *survminer* packages and compared with the log-rank test.

Since the expression of the EMILIN2 gene was shown to be down-modulated by methylation in a number of epithelial cancers [19], we first evaluated if the same epigenetic downregulation could also take place in melanoma patients. To this end, we queried the skin cutaneous melanoma (Firehose Legacy) TCGA cohort and drew the methylation landscape of the EMILIN2 gene using MEXPRESS (Figure S1). We verified that EMILIN2 also undergoes epigenetic modifications in this cancer type and, notably, some methylation sites were predominant in melanoma as opposed to normal skin (Figure 1A). Among these sites, we found that the methylation at site cg21266975 correlates with the overall survival of melanoma patients, with a lower methylation being protective and associated with a better outcome (Figure 1B). We also verified that the methylation of the EMILIN2 gene results in lower gene expression levels. Although not significant, there was a trend that high EMILIN2 expression levels were associated with increased overall survival in two different melanoma patient cohorts (Figure 1C and Figure S2).

**Figure 2:**
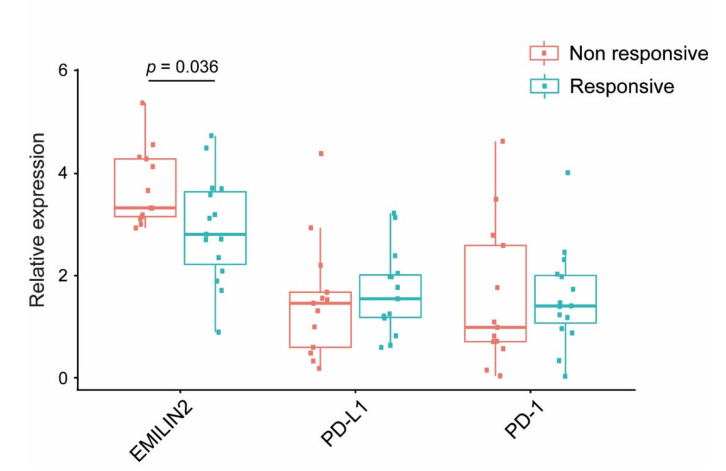
The EMILIN2 mRNAexpression levels associated with the response to α-PD-L1 therapy in melanoma patients. Analyses of the expression levels of the EMILIN2, PD-L1 and PD-1 genes in responder and non-responder melanoma patients upon PD-L1 blockage, as assessed in the GSE782209 cohort.

Published preclinical observations indicate that EMILIN-2 regulates vascular efficiency, thus impacting drug delivery [19]. To determine whether EMILIN2 could predict the therapeutic efficacy in melanoma patients treated with checkpoint inhibitors, we analyzed the EMILIN2 levels in a well-documented cohort of melanoma patients treated with PD-L1 inhibitors (GSE782209) [24]. In these patients, the expression levels of PD-1 and PD-L1 were comparable between the responder and non-responder patients (Figure 2), whereas the expression of EMILIN2 was significantly lower in responders compared to the non-responders (Figure 2), suggesting that it could be associated with immunotherapy efficacy. Notably, a significant inverse correlation between the expression of the EMILIN2 and PD-L1 genes was found in non-responder patients, whereas no correlation was observed in the responders (Figure S3), possibly due to the very low levels of EMILIN2 in this group of patients.

### 2.2. Loss of Emilin2 Is Associated with Improved Efficacy of PD-L1 Blockage

**Figure 3:**
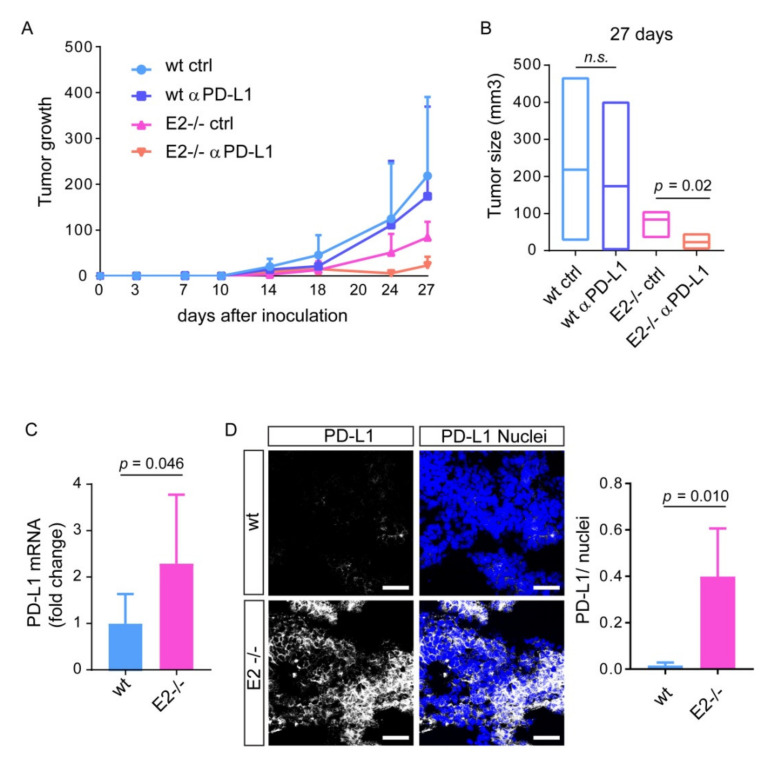
E *Emilin*2 null mice display improved response to anti-PD-L1 treatment. (**A**) Growth curve of B16F10 tumors injected in wild type (wt) and *Emilin2^−/−^*(E2^−/−^) mice and treated with anti-PD-L1 blocking antibody (αPD-L1) or isotype control (ctrl). *n* = 5 mice per group. (**B**) Tumor size 27 days following injection of B16F10 in mice as in A. Lines represent the median value per group. (**C**) Relative mRNA expression of PD-L1 in B16F10 tumors grown in wild type (wt) and *Emilin2^−/−^*(E2^−/−^) mice. *n* = 5 mice per group. (**D**) Representative images (**left**) and quantification (**right**) of the PD-L1 (white) signal in melanomas developed in wild type (wt) and *Emilin2^−/−^*(E2^−/−^) mice. Blue: nuclei; scale bar = 100 μm. *n* = 5 mice per group. n.s.–not significant. Graphs represent the mean ± SD. *p-*values were obtained using the paired Student’s t-*t*est.

To explore the putative association between EMILIN2 and the efficacy of PD-L1 inhibition, we took advantage of the *Emilin*2 null mouse. To this end, we subcutaneously injected wild type and *Emilin2^−/−^* animals with B16F10 syngenic melanoma cells and treated them with an anti-PD-L1 blocking antibody or the isotype control and monitored tumor growth over time (Figure 3A). As expected given the pro-angiogenic role of EMILIN2 [20], tumors grew more efficiently in wild type than in *Emilin2^−/−^* animals (Figure 3A). Interestingly, the administration of the anti-PD-L1 antibody reduced tumor growth by 20.3% in wild type mice compared to the control (Figure 3B), whereas the efficacy of the treatment was considerably superior in *Emilin2^−/−^* mice, accounting for a 72.7% reduction inthe tumor growth (Figure 3B).

Given the higher efficacy of PD-L1 blockage in *Emilin2^−/−^* mice, we assessed if the expression of PD-L1 and PD-1 was dissimilar in the two mouse models. Indeed, while the PD-1 mRNA levels were comparable (Supplementary Figure S4A), the expression of PD-L1 was higher in tumors developed in *Emilin2^−/−^* animals (Figure 3C). This finding was further confirmed by immunofluorescence analyses indicating that the PD-L1 protein levels were 25 fold higher in tumors developed in *Emilin2^−/−^* mice compared to wild type-derived tumors (Figure 3D).

### 2.3. The Blockage of PD-L1 Promotes Tumor Angiogenesis and Pericytes Recruitment Rescuing the Vascular Defects in Emilin2 Null Mice

**Figure 4:**
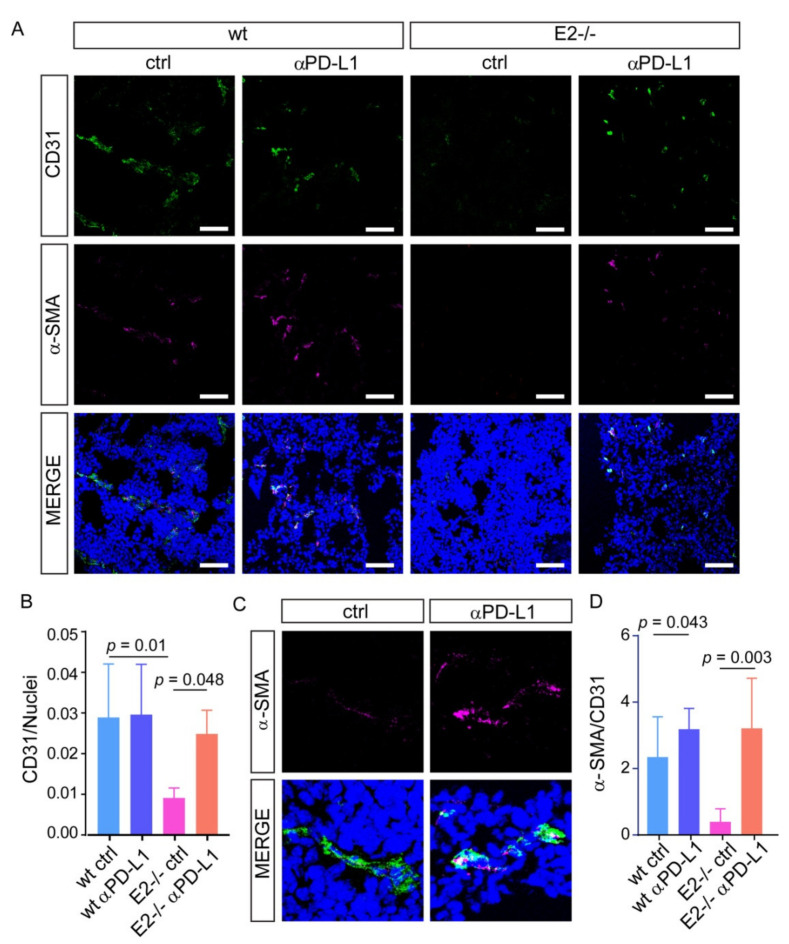
PD-L1 blockage induces vessel normalization in tumors grown in *Emilin2^−/−^* mice. (**A**) Representative images of CD31 (green, endothelial cells) and α-SMA (magenta, pericytes) staining performed on melanomas developed in wild type (wt) and *Emilin2^−/−^* (E2^−/−^) mice treated with the anti-PD-L1 blocking antibody (αPD-L1) or the isotype control (ctrl). Blue: nuclei; scale bar = 100 μm. *n* = 5 mice per group. (**B**) Quantification of the relative CD31 staining in tumors from the experiment reported in A. (**C**) Representative images of the CD31 (green) and α-SMA (magenta) staining performed on melanomas developed in *Emilin2^−/−^* (E2^−/−^) mice treated with anti-PD-L1 blocking antibody (αPD-L1) or isotype control (ctrl). Blue: nuclei. (**D**) Quantification of α-SMA staining relative to CD31 in tumors as in A. *n* = 5 mouse per group. Graphs represent the mean ± SD. *p* values were obtained using the one-way ANOVA Test.

**Figure 5:**
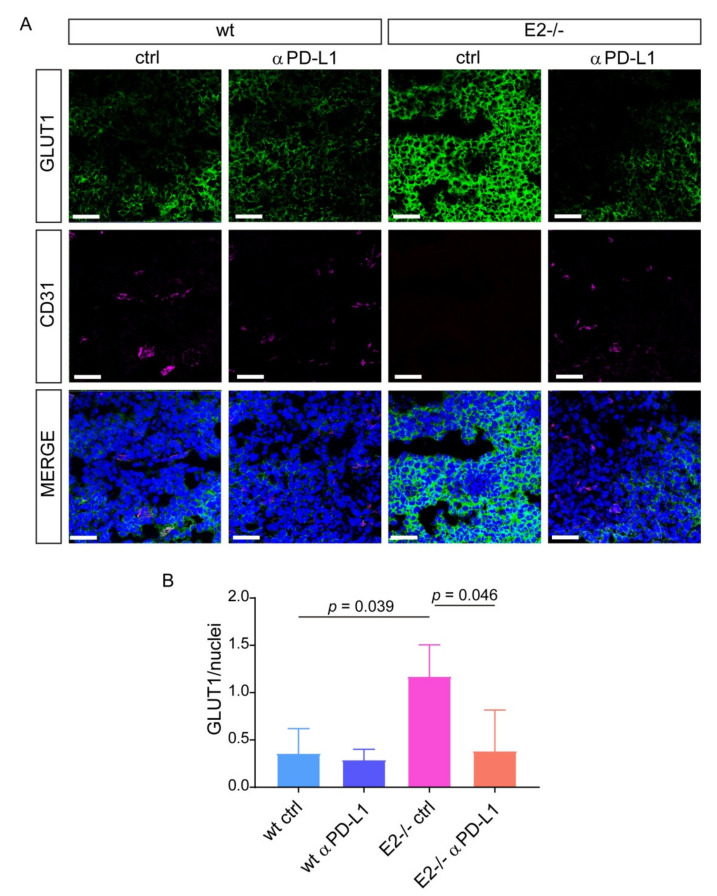
The blockage of PD-L1 is associated with reduced tumor hypoxia. (**A**) Representative images of GLUT1 (green) and CD31 (magenta) staining performed on melanomas developed in wild type (wt) and *Emilin2^−/−^*(E2^−/−^) mice treated with the anti-PD-L1 blocking antibody (αPD-L1) or the isotype control (ctrl). Blue: nuclei; scale bar = 100 μm. *n* = 5 mice per group. (**B**) Quantification of the relative GLUT1 staining in tumors as in A. *n* = 5 mice per group. Graphs represent the mean ± SD. *p* values were obtained using the one-way ANOVA Test.

It is well known that vessel normalization can improve the efficacy of immune checkpoint blockade; however, less is known about the impact of PD-1/PD-L1 inhibition in tumor vascularization. We verified the effect of the treatment in *Emilin2^−/−^* mice, characterized by altered vascularization, lower numbers of vessels, poor perfusion and impaired pericyte coverage. Strikingly, the anti-PD-L1 treatment in *Emilin2^−/−^* mice, which was conducted for 20 days, triggered tumor vascularization, as assessed by CD31 staining (Figure 4A,B). Notably, PD-L1 inhibition not only promoted tumor vascularization, but was also associated with an increased recruitment of pericytes to levels comparable with those observed in wild type animals, as assessed by α-SMA staining (Figure 4C,D). Importantly, the vascular changes induced by the treatment improved the efficiency of these vessels, ameliorating oxygenation of the tumors, as demonstrated by the analysis of GLUT1, a marker of hypoxia (Figure 5A,B).

### 2.4. Loss of EMILIN-2 in the TME Is Associated with Altered PD-L1 Expression in Melanoma Cells

**Figure 6:**
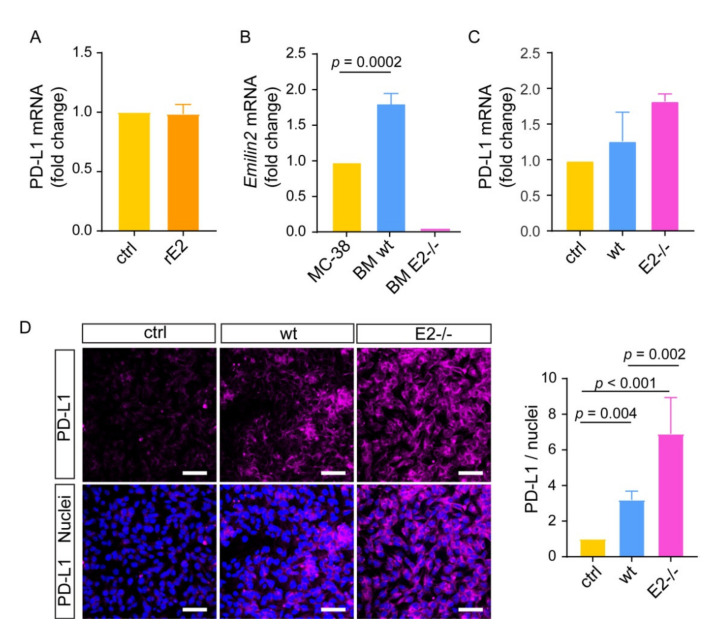
EMILIN-2 does not directly affect PD-L1 expression in melanoma cells. (**A**) Relative PD-L1 expression levels in B16F10 cells challenged with recombinant EMILIN-2 (rE2) or PBS (ctrl). (**B**) PCR evaluation of *Emilin2* expression by bone marrow derived cells isolated from wild type and *Emilin2*^−/−^ mice. (**C**) Relative PD-L1 mRNA expression levels in B16F10 cells co-cultured with bone marrow cells from wild type and *Emilin2*^−/−^ mice. (**D**) Representative images and relative quantification of the PD-L1 protein levels (magenta) as assessed by IF in B16F10 cells co-cultured as in (**C**). Blue: nuclei; scale bar = 50 μm. Graphs represent the mean ± SD. In (**B**–**D**), *p* values were obtained using the one-way ANOVA Test.

To verify if EMILIN-2 could directly impact PD-1/PD-L1 expression in the TME, we challenged B16F10 cells with recombinant EMILIN-2. EMILIN-2 did not affect the mRNA levels of PD-1 (Figure S4B). Similar results were obtained when analyzing the expression of PD-L1, both at the mRNA and protein levels, upon treatment of B16F10 with recombinant EMILIN-2 (Figure 6A and Figure S4C,D). These results suggested that the increased expression of PD-L1 depended on TME-mediated mechanisms elicited by EMILIN-2. Indeed, tumor cells are susceptible to microenvironmental stimuli that can induce the production of PD-L1; for instance, inflammatory cells were shown to trigger the expression of PD-L1 in B16F10 cells [25]. To verify if EMILIN-2 could impact PD-L1 expression through the involvement of immune cells, we established a co-culture of B16F10 and bone marrow cells extracted from wild type and *Emilin2^−/−^* mice. Of note, we verified that *Emilin*2 was efficiently expressed by bone marrow cells derived from wild type animals, whereas, as expected, no expression was detected in cells derived from *Emilin2^−/−^* mice (Figure 6B). In addition, bone marrow cells derived from wild type and *Emilin2^−/−^* mice displayed similar mRNA expression levels of PD-L1 (Figure S4F). On the contrary, while the PD-1 mRNA expression did not change (Figure S4E), the expression of PD-L1 was higher in B16F10 cells co-cultured with bone marrow cells derived from *Emilin2^−/−^* mice (Figure 6C,D). Taken together, these results suggested that EMILIN-2 could be implicated in the regulation of PD-L1 expression by tumor cells and may be exploited to predict the response to immunotherapy.

### 2.5. EMILIN2 Improves the Prediction Performance of Gene-Based Signatures

**Figure 7:**
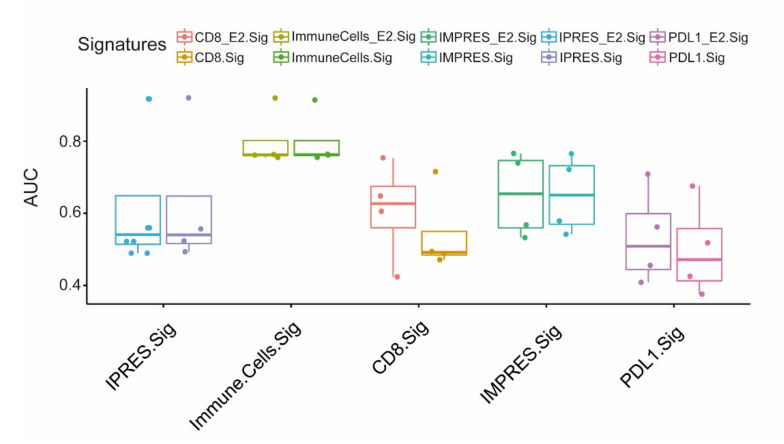
EMILIN2 improves the predictive value of gene expression-based signatures. Evaluation of the AUC upon the addition of EMILIN2 to the indicated gene signatures. Although not significant, these data suggest that the inclusion of EMILIN2 may substantially improve the median differences of some signatures.

Gene expression signatures have the potential to improve the prediction of the biological behavior of melanoma by objectively identifying the “high risk” patients [26]. To validate the value of EMILIN2 as a biomarker to identify the putative responders to immunotherapy, the evaluation of the EMILIN2 gene expression was added to previously published predictive signatures. To this end, we analyzed three independent gene expression datasets of melanoma patients (GSE91061, PRJEB23709, and MGSP datasets) as described by Xiong D. and co-authors [27]. The ROC analysis showed no increase in terms of area under the curve(AUC) when EMILIN2 was included in gene signatures comprising a large number of genes, i.e., IMPRES and IPRES, whereas the inclusion of EMILIN2 could significantly improve the predictive value of signatures comprising a restricted number of genes, i.e., the CD8 and PD-L1 signatures (Figure 7). Taken together, these results demonstrate that the expression of EMILIN2 can impact the efficacy of the treatments affecting both vascular efficiency and immunotherapy.

## 3. Discussion

Based on preclinical animal studies showing that the inhibition of the PD-1/PD-L1 signaling axis could restore the function of exhausted T-cells, thus re-establishing the antitumor activity of the immune system, several PD-1/PD-L1 blocking antibodies have been developed. Immune checkpoint inhibitor-based immunotherapy radically improved the management of melanoma patients, and it is now considered one of the most promising approaches in the combat cancer [28]. Nonetheless, the failure of anti-PD-1/PD-L1 immunotherapies to sustain a durable response in some melanoma patients emphasizes the need to delineate the tumor resistance mechanisms, characterize biomarkers for patient selection, and develop rational combinatorial therapeutic strategies. Emerging evidence suggests that the ECM and its remodeling products exert a crucial role in the regulation of the cancer-immunity cycle, and impact the mechanisms of resistance to immunotherapy [29]. In this view, the ECM proteins represent a promising therapeutic target and a route to discover new biomarkers that may be of value for the management of cancer patients [30]. In the present work, we showed that the expression of EMILIN2, which in melanoma is regulated by methylation, is variable among the patients and associated with the efficacy of anti-PD-L1 therapy. Moreover, by analyzing a well-established preclinical murine melanoma model, we demonstrated that *Emilin2* serves as a regulator of PD-L1 expression. Importantly, we found that the treatment with an anti-PD-L1 inhibitor led to the normalization of the exceedingly abnormal vasculature characterizing *Emilin2^−/−^*-derived tumors, with an increased number of vessels, a higher pericyte coverage and overall reduced intra-tumor hypoxia. Few sources describing the role of PD-L1 inhibition on tumor vessel normalization have been published [31] and, to our knowledge, this is the first time that this effect is found in melanoma. It is possible to speculate that the altered angiogenesis due to EMILIN2 loss could be overtaken by the activation of Th1 lymphocytes which, in turn, among other cytokines, secrete IFN-γ, with a consequent impact on melanoma-associated vascular normalization, as previously demonstrated [31]. These observations further support the notion that the immune and vascular systems are tightly interconnected and that combining anti-angiogenic and immune therapies may represent a promising approach to improve the survival of the patients [32]. The identification of subgroups of patients that will most likely benefit from immunotherapy is an ongoing challenge, as indicated by the great deal of effort that has been made to draw expression-based signatures with predictive value. Notably, in this study, we found that the analysis of the EMILIN2 gene may be useful for the identification of the responders to the treatment with checkpoint inhibitors. In particular, we verified that the inclusion of EMILIN2 ameliorates the AUC median of the CD8 and PDL1 signatures. Despite the difference not being significant, it is interesting to point out that the median difference improves upon addition of EMILIN2; however, further studies are needed to confirm these results. The same increment was not observed in already rich and complex signatures, which are able to describe many features of the tumor microenvironment, but, on the other end, are costly and hardly applicable in clinical practice.

We envision that, due to the multiple roles exerted by EMILIN-2 in the tumor microenvironment, the molecule has potential value as a good biomarker to predict the efficacy of PD-L1 blockade strategies. Similarly to the other members of the family, the molecular structure of EMILIN-2 is very complex and arranged in different domains [33], endowing the glycoprotein with the capacity to interact with many receptors and secreted proteins/growth factors, thus regulating many functions within the tumor microenvironment. In this context, the pro-angiogenic function of EMILIN-2 is of particular interest. Indeed, low levels of EMILIN-2 not only imply reduced tumor vascularization but also impaired vascular efficiency, which, in turn, leads to poor drug delivery within the tumors [20]. On the other hand, in this study, we demonstrate that low EMILIN-2 levels are associated with increased PD-L1 expression in melanoma and with improved immunotherapy efficacy. PD-L1 expression in tumor cells is induced by a variety of growth factors including INF-γ, IL-1α and IL-27 [34]. Since our results indicate that EMILIN-2 does not directly affect the expression of PD-L1 in melanoma cells, it is conceivable that it could suppress the production of such cytokines in immune cells. This possibility is also suggested by the increased production of the checkpoint molecule in B16F10, when co-cultured with bone marrow cells derived from *Emilin2^−/−^* mice. As to whether the above-mentioned cytokines are involved in this indirect stimulation, further investigation isneeded, as well as identification of the molecular pathways activated in immune cells. Interestingly, in this context, IFN-γ may represent the key cytokine through which EMILIN-2 impacts both vascular efficiency and immunotherapy efficacy. In addition to IFN-γ, we have previously demonstrated, in a number of cell types [20,23], that EMILIN-2 elicits the expression of IL-8, a paradigmatic cytokine in the crossroads between angiogenesis and inflammation [35,36,37], and that the low expression of EMILIN-2 characterizing some melanoma patients may result in altered activation of the two processes. Moreover, given that the EMILIN-2-dependent IL-8 production is driven by the activation of the EGF/EGFR signaling pathway, it is conceivable to speculate that this signaling pathway may be taken into account to draw new combination therapies.

Our previous investigations indicate that low levels of EMILIN-2 are associated with exacerbated tumor progression, since, as mentioned, EMILIN-2 exerts tumor suppressive functions overall [15,16,17]. However, this may depend on the tumor type, as well as the specific microenvironment. It must be pointed out that, in our opinion, this is not surprising. In fact, the anti-tumoral activity exerted by EMILIN-2 during tumor progression may be overcome during treatment. This is particularly likely when employing drugs with biological activity such as checkpoint inhibitors, since their action may be affected by the microenvironmental features characterizing each individual, thus resulting in diverse efficacy in the different patients. Given the role of EMILIN-2 in controlling vascular efficiency, this concept can also be extended to conventional chemotherapy, since the distribution of the drugs may be more impaired in patients displaying low levels of EMILIN-2. Indeed, EMILIN-2 may represent a key molecule in the cross-talk between inflammation and angiogenesis, which represents a new therapeutic frontier [38].

Taken together, the results provided in this study suggest that patients characterized by a highly methylated EMILIN2 gene may display poorly efficient vasculature that may be associated with reduced drug delivery to the tumor, and, on the other hand, by an increased immune-suppressive TME. Nonetheless, according to our pre-clinical results, patients characterized by low EMILIN-2 expression may be good candidates for immune checkpoint blockade, since the efficacy of the treatments improved significantly upon EMILIN-2 depletion. We envision that EMILIN-2 may be considered as a microenvironmental tool that could be exploited to define important features of the TME and to better delineate therapeutic strategies for melanoma patients.

## 4. Materials and Methods

### 4.1. Antibodies and Reagents

The human monoclonal 828B3B3 and polyclonal anti-EMILIN-2 antibodies were produced in our laboratories as previously described [15,17]. The anti-histidine antibody was from Abgent (San Diego, CA, USA). Ni-NTA agarose was purchased from QIAGEN (Milan, Italy). The secondary Alexa Fluor 488- and 546-conjugated antibodies and TO-PRO- 3 were from Invitrogen (Milan, Italy). The monoclonal anti-human CD31 antibody was from Invitrogen (Milan, Italy), the anti-αSMA antibody was purchased from Abcam (ab5694, Abcam, UK) and the anti-GLUT1 from Merck Millipore (Milan, Italy). The FuGene6 reagent was purchased from Promega (Milan, Italy). The in vivo anti-mouse PD-L1 antibody and the rat IgG2bisotype control were from BioXcell (Lebanon, NH, USA).

### 4.2. Cell Cultures

The 293-EBNA and B16F10 melanoma cell lines were obtained from ATCC (Manassas, VA, USA). B16F10 and 293-EBNA cells were cultured in Dulbecco’s modified eagle’s medium (DMEM) with 10% fetal bovine serum FBS (Gibco, Milan, Italy). All cells were maintained at 37 °C under a humidified atmosphere containing 5% CO_2_ and verified to be free of mycoplasma contamination using the MycoAlert™ Mycoplasma Detection kit from LONZA (Basel, Switzerland).

For recombinant EMILIN-2 production, E293 cells were transfected using the FuGene6 reagent according to the manufacturer’s instructions. E293 cells transfected with the pCEP-Pu-EMILIN2 constructs were selected with 250 μg/mL of G418 and 0.5 μg/mL of puromycin. Confluent E293 cells were then incubated in serum-free medium for 48 h, following by media collection and protein purification with Ni-NTA beads.

For PD-L1 expression analysis, B16F10 melanoma cells were challenged with recombinant EMILIN-2 (3 µg/mL) or PBS as negative control. After 48 h, mRNA was extracted with Trizol and processed as described in Section 4.5.

### 4.3. Isolation of Bone Marrow-Derived Cells and Co-Cultures with B16F10

The bone marrow was isolated from murine tibias and femurs as previously described [39]. Red blood cells were lysed in ammonium chloride potassium buffer for 4 min. B16F10 cells were stained with the fluorescent DiI dye to distinguish them in the co-cultured with unlabeled bone marrow-derived cells; the cells were plated at a 1:10 ratio. Cells were maintained in RPMI/F-12 medium (Gibco, Milan, Italy) at a ratio of 1:1 for 48 h and, after that, the RNA was collected and coverslips were fixed for further analyses.

### 4.4. Immunofluorescence

For immunofluorescence analyses on tumor samples, serial cryostatic 7μm sections were collected on positively charged slides (BDH Superfrost Plus, Leica Microsystems Heidelberg, Mannheim, Germany) and air dried at room temperature (RT) following a fixation with PFA for 15min. Slices were incubated with 0.5% Triton X-100 in PBS for 5 min at RT and blocked with 2% BSA in PBS for 1hr at RT. Primary antibodies were incubated overnight at 4 °C followed by incubation with fluorescently conjugated secondary antibodies for 1h at RT. Sections were mounted in Mowiol containing 2.5% (*w/v*) of 1,4-diazabicyclo-(2,2,2)-octane (DABCO) and images were acquired with a Leica TCS SP8 Confocal system (Leica Microsystems Heidelberg, Mannheim, Germany), using the Leica Confocal Software (LCS). Fluorescence intensity and quantification was evaluated by means of the Volocity software (PerkinElmer Inc., Waltham, MA, USA).

### 4.5. RT-qPCR

Total RNA was isolated from cell lines with Trizol and reverse transcribed using AMV-RT (Promega, Milan, Italy). After determination of the primer specificity and efficiency, Real-time RT-PCRs were performed with iQTM SYBR^®^ Green Supermix (Bio-Rad, Milan, Italy) and BIORAD CFX96 TouchTM Real Time PCR Detection System. The oligonucleotides used are listed in Table 1.

The specificity of the Real-time RT-PCR reactions was determined by analyzing the melting curve of the amplified products, which were evaluated by agarose gel electrophoresis, and the 2^−ΔΔCt^ method could be applied for the analyses.

### 4.6. In Vivo Tumor Growth

Six weeks old wild-type and *Emilin2*^−/−^ C57B6N mice were subcutaneously injected in both flanks (5 × 10^5^ cells/flank) with B16F10 melanoma cells resuspended in 100 μL of DMEM without phenol red (Gibco, Milan, Italy). The treatment with the anti-PD-L1 and the anti-IgG2b isotype control antibodies started 7 days following the cell injection and was performed twice a week until the end of the experiment. Tumor growth was measured every 2 days using caliper. At the end of the experiment, tumors were excised and included in OCT for subsequent immunofluorescence analyses. All the in vivo studies were approved by the Institutional Ethics Committee of the CRO of Aviano and the Italian Ministry of Health (project # 148/2016-PR).

### 4.7. Bioinformatics and Statistical Analysis

The RNA sequencing data, somatic mutation data, clinicopathological and survival data of Melanoma cancers (SKCM) were downloaded from UCSC Xena (https://xenabrowser.net, accessed on 12 March 2021). MEXPRESS was used to evaluate methylation of EMILIN2 probes on clinical data (http://mexpress.be, accessed on 12 March 2021). EMILIN2 expression was evaluated in two metastatic datasets using cbioportal [40,41]. To validate this role of EMILIN2 gene as biomarker, we analyzed 4 independent gene expression datasets of melanoma patients (GSE78220, GSE91061, PRJEB23709, and MGSP datasets) recently used in an article by Xiong et al. [27]. All the analyses were developed using R (4.0) on a cluster computer.

Statistical analyses were performed with the SigmaPlot and GraphPad Prism 6 software (Graphpad) and the values represent the mean ± standard deviation obtained with at least three measurements on randomized samples. The statistical significance of the differences was determined by the two-sided Student’s t-test for the comparisons between two groups; for more than two groups, the ANOVA 1-way analysis of variance was used, according to the Bonferroni method. For the in vivoexperiments, mice were randomly assigned to treatment groups. Differences were considered statistically significant when *p* ≤ 0.05.

## Figures and Tables

**Table 1:** List of the oligos employed.

Gene	Oligonucleotide
CD31 For	5′-TGTCATTGGAGTGGTCATCG-3′
CD31 Rev	5′-TGTTGGAGTTCAGAAGTGGAG-3′
PD-L1 For	5′-GGAATTGTCTCAGAATGGTC-3′
PD-L1 Rev	5′-GTAGTTGCTTCTAGGAAGGAG-3′
PD-1 For	5′-TTCAGGTTTACCACAAGCTGG-3′
PD-1 Rev	5′-TGACAATAGGAAACCGGGAA-3′
Actin For	5′-CTGTCGAGTCGCGTCCACC-3′
ActinRev	5′-ATCGTCATCCATGGCGAACTG-3′
*Emilin2* For	5′-CCCAGTGCCAGGAACAAAA-3′
*Emilin2* Rev	5′-AATAAAACTCGCTTCCCTC-3′

## Data Availability

Not applicable.

## Supplementary Materials

The following are available online at https://www.mdpi.com/article/10.3390/ijms22147511/s1.

## Abbreviations

MDPI	Multidisciplinary Digital Publishing Institute
ECM	Extracellular matrix
TME	Tumor microenvironment
CAF	Cancer-associated fibroblasts
PD-1	Programmed cell death protein-1
PD-L1	Programmed death-ligand-1
EGF	Epithelial growth factor
EGFR	Epithelial growth factor receptor
TCGA	The cancer genome atlas
CD31	Cluster of differentiation -31
CD8	Cluster of differentiation -8
AUC	Area under the curve
α-SMA	Alpha-smooth muscle actin
IL-8	Interleukin-8
