# Supplementary material for: The Efficacy of Anti-PD-L1 Treatment in Melanoma Is Associated with the Expression of the ECM Molecule EMILIN2
Source: Int J Mol Sci. 2021 Jul 13;22(14):7511. doi: 10.3390/ijms22147511 (PMC8306837; doi:10.3390/ijms22147511)
Supplement: Supplementary file 1 [file ijms-22-07511-s001.zip › ijms-1248627-supplementary.pdf]

Supplementary Figure 1

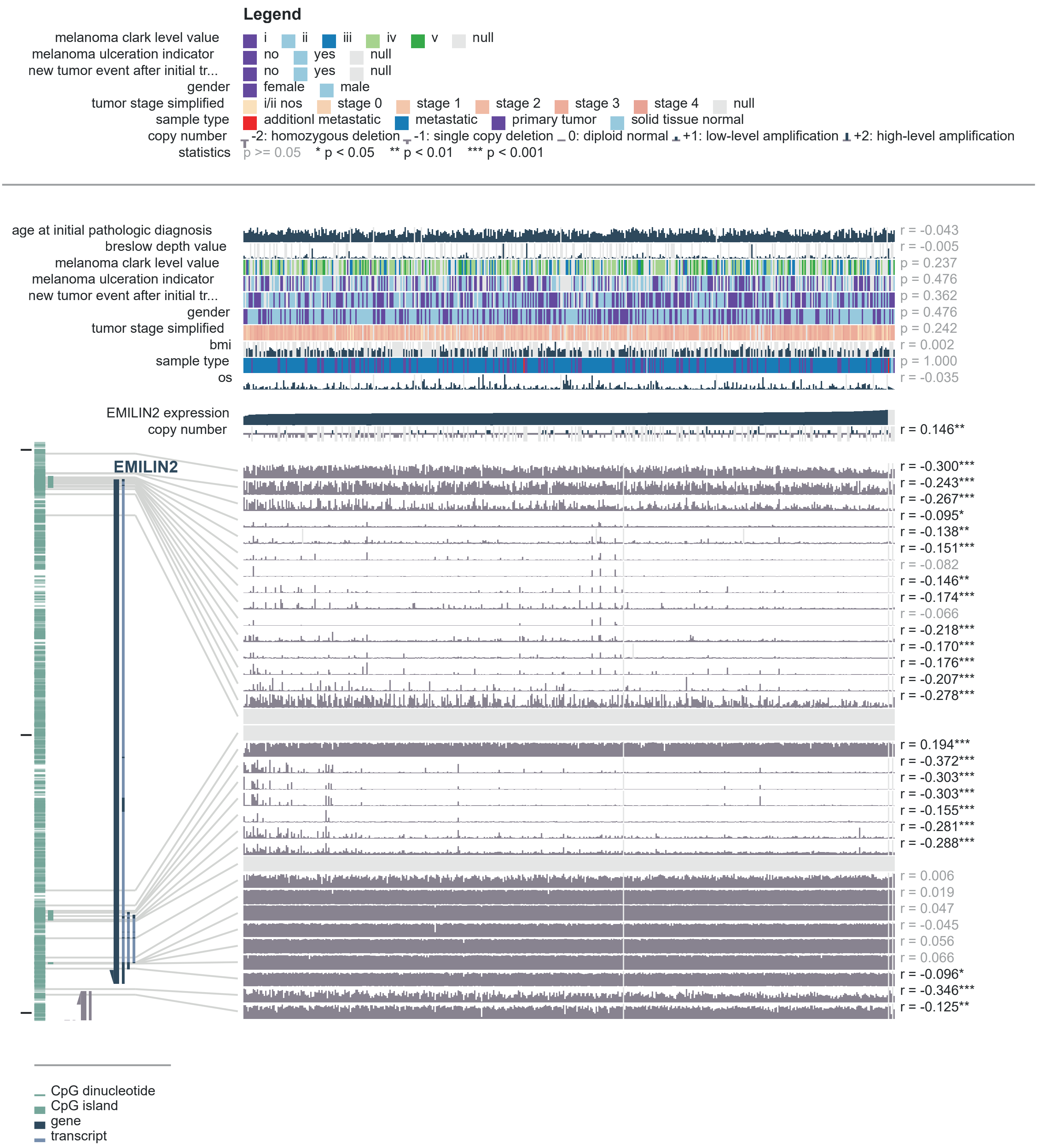

Supplementary Figure S1. EMILIN2 gene is methylated in melanoma samples. Epi -genetic landscape of EMILIN2 gene and clinical /molecular parameters as assessed by MEXPRESS (accessed on 12 March 2021). The analyses are in relation to EMILIN2 levels and samples ordered based on the expression value. The Pearson correlation coefficients are reported on the right and highlight a significant correlation between the expression of the EMILIN2 gene, the methylation probes and the corresponding clinical parameters.

Supplementary Figure 2

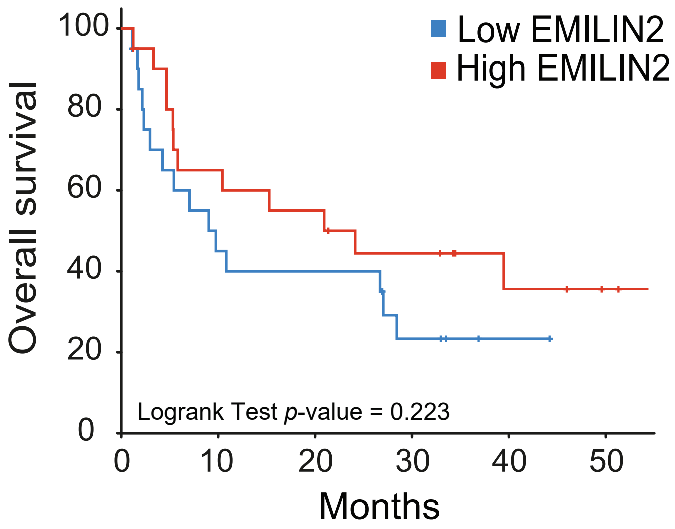

Supplementary figure S2. EMILIN2 associates with overall survival. Kaplan-Meiercurvestratifiedaccordingtohighandlow EMILIN2expressionlevelsina cohort of melanoma patients (Van Allen et al, 2015). Kaplan-Meier curves were computed using R (version 3.6.1) with survival and survminer packages and compared with the log-rank test.

Supplementary Figure 3

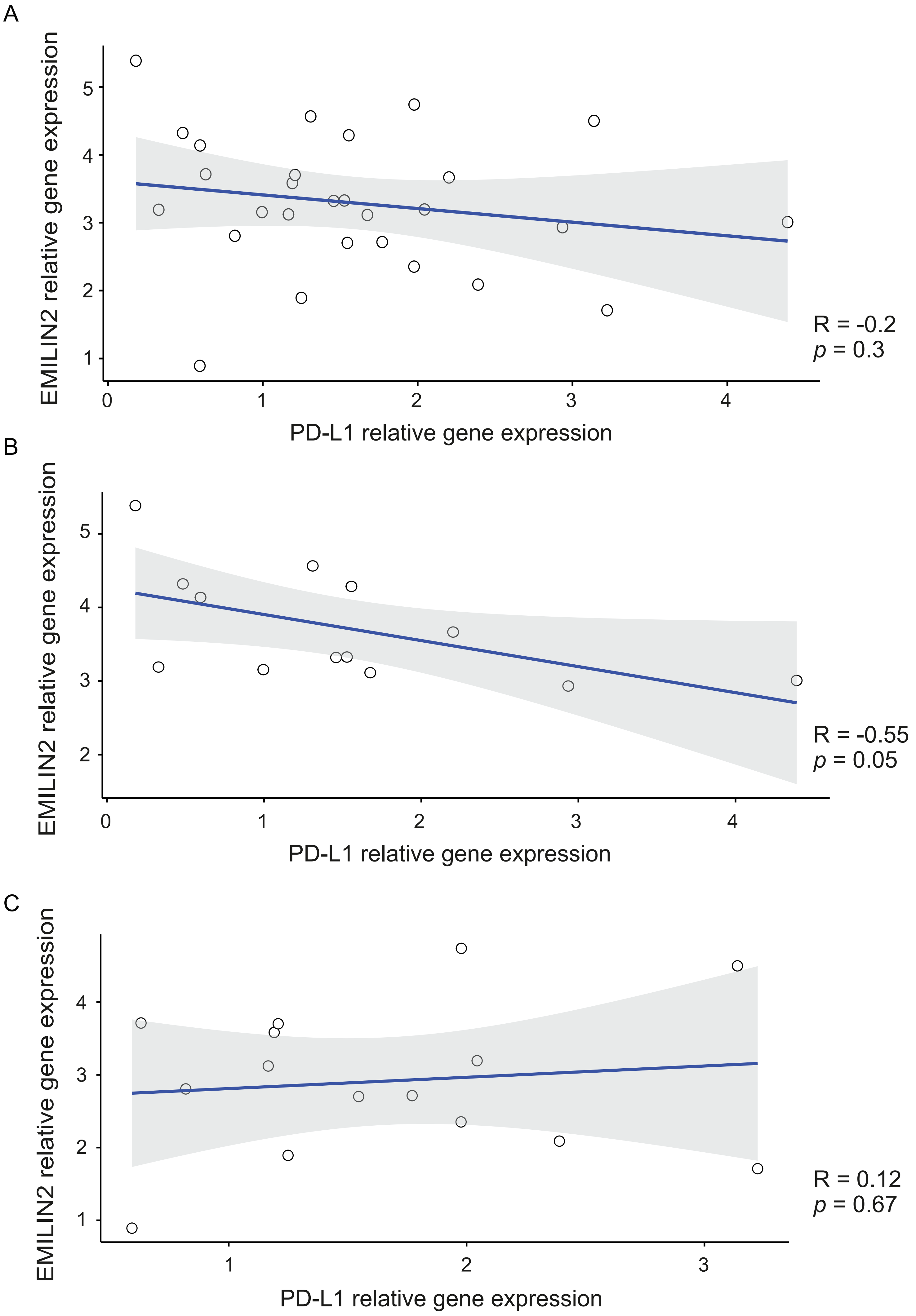

Supplementary Figure S3. The EMILIN2 gene expression inversely correlates with that of PD-L1 in non-responder patients. A-C) Pearson correlation analyses between the expression of the EMILIN2 and PD-L1 genes in all patients (A), non-responders (B) and responders (C).

Supplementary Figure 4

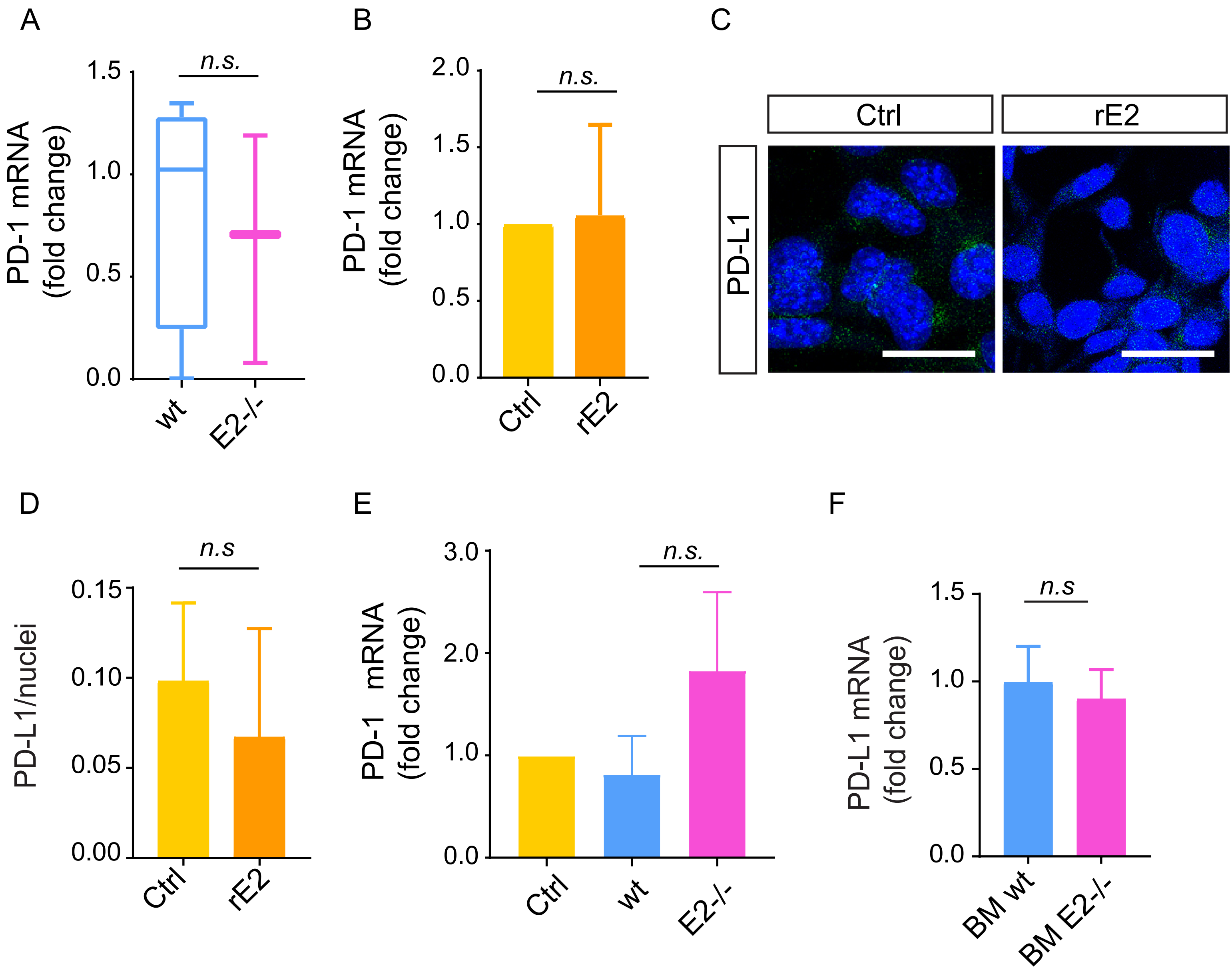

Supplementary Figure S4. Expression of PD-1 and PD-L1 in relation to EMILIN-2. A) Re-lative mRNA expression level of PD-L1 in B16F10 tumors grown in wild type (wt) and *Emi-lin2*$^{-/-}$ (E2-/-) mice. n = 5 mice per group. *P* value was obtained using the paired Student's t-test. B) Relative mRNA expression level of PD-1 in B16F10 cells challenged in vitro with recombinant EMILIN-2 (rE2) or PBS (ctrl). *P* value was obtained using the paired Student's t-test. C) Representative images of the immunofluorence analyses of PD-L1 (green) in B16F10 cells treated with PBS (Ctrl) or recombinant EMILIN-2 (rE2),nuclei are stained with TO-PRO (blue). Scale bar = 25 μm. D) Graph representing quantitative analyses of the resul-ts reported in C. E) Relative expression levels of PD-1 in B16F10 cells co-cultured with bone marrow cells isolated from wild type (wt) and *Emilin2*$^{-/-}$ (E2-/-) mice; B16F10 alone were used as a control (Ctrl). n = 5 per group. *P* values were obtained using the one-way ANOVA Test. Graphs represent the mean ± SD. F) Graph representing the relative expression of PD-L1 in bone marrow cells isolated from wild type (BM wt) or *Emilin2*$^{-/-}$ (BM E2-/-) mice. *P* value was obtained using the paired Student's t-test.
